# Family planning awareness among never-married adolescent girls in Bangladesh

**DOI:** 10.1371/journal.pone.0356778

**Published:** 2026-10-08

**Authors:** Sumaiya Nusrat, Md Tazvir Amin, Md. Mahabubur Rahman, Mohammad Hridoy Patwary, MHM Imrul Kabir, Shusmita Hossain Khan, M. Moinuddin Haider

**Affiliations:** 1 Maternal and Child Health Division, International Centre for Diarrhoeal Disease Research, Bangladesh (icddr, b), Dhaka, Bangladesh; 2 Health Systems and Population Studies Division, International Centre for Diarrhoeal Disease Research, Bangladesh (icddr, b), Dhaka, Bangladesh; 3 Department of Mathematical and Physical Science, East West University, Dhaka, Bangladesh; 4 Independent Consultant, Dhaka, Dhaka Division, Bangladesh; Jimma University, ETHIOPIA

## Abstract

**Background:**

This study aims to explore the national level prevalence of family planning (FP) awareness among never-married adolescent girls (NMAGs) in Bangladesh and perform a policy analysis to evaluate the effectiveness of existing FP programs and initiatives specifically designed to increase FP awareness among NMAGs in the country.

**Methods:**

We used data from the Bangladesh Adolescent Health and Wellbeing Survey (BAHWS) 2019–20, the first national-level cross-sectional survey conducted on adolescents aged 15–19 in Bangladesh. Bivariate and multivariable logistic regression models were employed to identify the correlates of FP awareness. The FP programs and initiatives taken since 2012 were assessed using the Center for Disease Control and Prevention (CDC) policy analysis framework.

**Findings:**

Among NMAGs, roughly 25% were unaware of any modern FP method, 28% were unaware of oral contraceptive pills, 60% did not know about condoms, and 91% were unfamiliar with emergency contraceptive pills. Younger adolescents, those with lower levels of education, living in rural areas or Central and Eastern divisions, without access to mobile phones or the internet, with limited exposure to media, and not recently exposed to adolescent programs, were less likely to know about modern family planning methods. Using CDC analysis, we found that no intervention or strategies have been specifically developed to increase FP awareness among NMAGs in Bangladesh.

**Conclusion:**

FP awareness among NMAGs shows pronounced socio-demographic disparities. Findings underscore the need for coordinated government–development partner collaboration to design and implement tailored, adolescent-responsive interventions and strategies to improve FP awareness among NMAGs and safeguard their Sexual and Reproductive Health and Rights.

## 1. Introduction

Family planning (FP) is a fundamental aspect of reproductive health, aimed at empowering individuals to make informed decisions about birth spacing and achieving their desired number of children [1]. Over the years, cohabiting couples across the globe have increasingly adopted FP as an essential part of reproductive health, recognizing its effectiveness in supporting informed decisions about birth timing and spacing, improving maternal and child health, and enhancing overall family well-being [2,3]. Effective use of FP can also avert the 73 million induced abortions occurring every year globally [4,5].

Despite the progress in FP uptake among adult populations, particularly those in marital or cohabiting unions, early understanding and awareness of FP during adolescence remain limited in many parts of the world. Globally, only 31.6% of adolescent girls use modern contraception, and over half (50.8%) have an unmet need for family planning, indicating significant gaps in both usage and awareness compared to older women [6]. Adolescence is a crucial developmental phase in which young people begin to form their values, attitudes, and behaviors related to sexuality and reproduction. Introducing FP awareness during this formative stage is essential, as it prepares adolescents to make informed decisions before they become sexually active or enter into marriage or unions [7]. Globally, a significant number of individuals initiate sexual activity during adolescence [8,9], with approximately 16 million adolescent pregnancies occurring each year [10]. A substantial proportion of these pregnancies are unintended and often end in unsafe abortions, posing serious risks to the physical and mental health of young individuals and contributing to broader social and economic challenges [11,12]. Without timely access to accurate information and services related to FP, adolescents are more vulnerable to negative reproductive health outcomes that can impact their long-term well-being, educational attainment, and future opportunities.

FP awareness among adolescents is crucial for making informed decisions about birth control, especially where child marriage is prevalent [13]. In this regard, the girls of low- and lower-middle-income countries (LLMICs) are more vulnerable to child marriage than the rest. In LLMICs, approximately 36% of girls are married before the age of 18, with 10% married before age 15 [14]. In LLMICs, adolescent girls are often forced into marriage without adequate reproductive health awareness, which leads to numerous unwanted pregnancies and abortions [15,16]. In addition, adolescent mothers (under the age of 20) have higher rates of obstructed labor, fistula, premature delivery, and low birth weight babies than older mothers, which also underscores the importance of knowing FP methods from adolescence [17]. However, limited information is available on the level of FP awareness among never-married adolescent girls (NMAGs) from LLMICs [18,19].

Bangladesh is the eighth most populous country in the world and a lower-middle-income country, which has been suffering from 38 million child marriages every year [20], where child marriage is disproportionately concentrated among girls from rural areas, poorer households, and those with lower levels of education [21]. As a result, the majority of these girls get married without prior awareness of FP, exposing them to the risk of unintended conception and induced abortion [22,23]. Over the years, the government of Bangladesh, NGOs, and development partners have launched several initiatives to promote sexual and reproductive health (SRH) and FP awareness among married adolescent girls (MAGs) [24]. However, these initiatives often overlook NMAGs [25].

The existing literature on adolescent reproductive health in Bangladesh is largely focused on MAGs or ever-married women, leaving a critical gap in understanding the situation of NMAGs. One study examining FP needs among MAGs in urban slums of Dhaka [26] did not address the specific FP awareness needs of NMAGs. Another study conducted in two low-performing sub-districts of Bangladesh [25] assessed reproductive health knowledge among unmarried adolescent girls but was geographically restricted, used a non-representative sample, and did not specifically measure FP method awareness as a primary outcome. At the global level, systematic reviews of contraceptive knowledge and use among adolescents in LMICs [18,19] do not disaggregate findings by marital status or provide Bangladesh-specific nationally representative data. Although a few studies have examined the level and correlates of FP awareness among MAGs in Bangladesh [13], no nationally representative study has specifically examined FP method awareness and its socio-demographic correlates among NMAGs in Bangladesh. The present study therefore aims to address this evidence gap by evaluating national-level FP awareness among NMAGs in Bangladesh.

The government of Bangladesh, in collaboration with development partners, has launched a few programs aimed at enhancing adolescent SRH awareness, raising FP awareness, and reducing the incidence of child marriage [27–32]. The Advancing Adolescent Health (A2H) program, BALIKA program, and Shukhi Jibon Project are recognized examples [29–31]. Policy formulation for improving FP awareness among NMAGs requires scrutinizing these programs. However, to the best author’s knowledge, no policy analysis has been conducted to assess the mentioned programs through the lens of enhancing FP awareness among NMAGs.

### 1.1. Objective

The objectives of this study are twofold:

- The primary objective of this study is to examine the level of FP awareness and its correlates among NMAGs aged 15–19 in Bangladesh.
- The second objective is to conduct a policy analysis to assess existing policy, programs, and strategies aimed at improving FP awareness among adolescents in Bangladesh.

## 2. Methodology

### 2.1. Data

This research is a secondary analysis of data from the Bangladesh Adolescent Health and Wellbeing Survey (BAHWS) 2019–2020 [33]. The survey was a nationally representative cross-sectional survey that collected data from ever-married adolescent girls, never-married adolescent girls, and never-married adolescent boys aged 15–19. For our analysis, we focused exclusively on the subset of 7,742 NMAGs.

### 2.2. Outcome measure

Participants were individually asked about their familiarity with contraceptive methods, with a specific focus on the question, “Which ways or methods have you heard about?”. A comprehensive list of contraceptive methods was presented during the inquiry. Responses falling under ‘spontaneous yes’ or ‘probed yes’ were grouped as “Yes” and coded as 1, while those indicating ‘probed no’ were recorded as “No” and coded as 0.

In this study, we conceptualize FP awareness as recognition-based awareness, defined as whether respondents have ever heard of specific contraceptive methods. This operationalization aligns with commonly used measures in large-scale surveys such as the Demographic and Health Surveys (DHS), where “hearing of a method” is considered an initial and essential step in the continuum of awareness and informed decision-making.

### 2.3. Covariates of interest

Based on the literature review, we grouped the correlates into three broad domains- individual characteristics, household characteristics, exposure to media, and adolescent-focused programs [34,35] (Table 1). Individual characteristics include respondents’ ages and educational attainment of the participants. Household characteristics include the respondent’s residence type (rural, urban), geographical locations within Bangladesh (western, central, or eastern), and wealth group. Exposure to media and exposure to adolescent programs includes mobile phone ownership, internet exposure, mass media access, and exposure to any adolescent-focused program during the three months preceding the survey.

**Table 1:** Description of covariates of interest among never-married adolescent girls in Bangladesh, 2019–2020.

*Domain*	*Factors*	*Description*
**Girl characteristics**	**Age**	
	15-17 years	Adolescents in mid-teenage years
	18-19 years	Late teenage years, close to adulthood
	**Years of schooling**	
	<5 years	less than primary completion
	>=5 years	At least primary completion
**Household characteristics**	**Type of residence**	
	Rural	Reside in rural area
	Urban	Reside in urban area
	**Region**	
	Western (Rangpur, Rajshahi, Khulna)	Western divisions of Bangladesh
	Eastern (Sylhet, Chattogram)	Eastern divisions of Bangladesh
	Central (Barishal, Dhaka, Mymensingh)	Central divisions of Bangladesh
	**Household wealth quintiles**	
	Lowest 20%	Lower class
	Second 20%	Lower middle class
	Middle 20%	Middle class
	Fourth 20%	Upper middle class
	Highest 20%	Upper class
**Exposure to media and adolescent-focused programs**	**Mobile access**	
	Own mobile phone/ access to family phone	The girl has her own mobile or access to a family phone
	No access to a mobile phone	The girl does not have her own mobile or access to a family phone
	**Internet access**	
	Yes	Using the internet through a mobile/computer
	No	Does not use internet
	**Mass media access**	
	TV, Radio, Newspaper (Yes)	Watch TV or listen radio, or read newspaper regularly
	TV, Radio, Newspaper (No)	Does not watch TV or listen radio, or read newspaper regularly
	**Exposure to adolescent program (last 3 months)**	
	Yes	Participated in one or more adolescent programs
	No	No participation in adolescent programs

**Note:** The household asset quintile is constructed by the household ownership of a set of assets as reported by the respondent or observed by the interviewer at the survey.

### 2.4. Statistical analysis

First, we conducted a univariate analysis to understand the characteristics of the sample. Then we conducted a bivariate analysis to explore the dependence of the outcome variables on covariate. The Chi-square test of association was performed to measure the strength of the dependence. Finally, we used multivariable logistic regression to examine the factors associated with awareness of FP methods. A complete case analysis approach was used to handle missing data. Multicollinearity was assessed using the variance inflation factor (VIF), and model fit was evaluated using F-statistics. Complex survey design was handled using the *svy* command in STATA. All analyses incorporated survey weights and were conducted using STATA version 14.2.

### 2.5. Program, policy, and strategy analysis

We considered the following relevant national guiding documents to understand the national programs, projects, and policy situation using the Center for Disease Control and Prevention (CDC) policy analysis framework.

- National Adolescent Health Strategy (2017–2030) (Ministry of Health and Family Welfare (MoHFW), the Directorate General of Health Services (DGHS), the Directorate General of Family Planning (DGFP)) [27].
- Bangladesh National Adolescent Strategy (2020) (MoHFW, Ministry of Education, Ministry of Social Welfare, Ministry of Women and Children’s Affairs (MoWCA)) [28].
- The Advancing Adolescent Health (A2H) program (2016–2019) (Implemented by Plan International and funded by the United States Agency for International Development (USAID)) [36].
- Shukhi Jibon Project (2018–2024) (Led by Pathfinder International, in collaboration with Bangladesh’s DGFP, DGHS, and NIPORT, funded by USAID) [37].
- The Bangladeshi Association for Life Skills, Income, and Knowledge for Adolescents (BALIKA) program (2012–2016) (Developed by Population Council, PSTC, Centre for International Development Issues Nijmegen, and mPower) [38].
- Engaging Volunteer Networks for Family Planning: Connecting Communities with Facilities (2015–2017) (Implemented by EngenderHealth, Funded by USAID) [39].

Improving the level of awareness regarding FP among NMAGs requires multisectoral policy responses. However, because this paper focuses on the level of awareness about FP among NMAGs aged 15–19 years and how it is associated with the relevant correlates, national policies directly linked with SRH and FP-focused adolescents were reviewed and analyzed.

#### 2.5.1. Centers for disease control and prevention (CDC) policy analysis framework.

*Domain 1: Problem Identification*

Step 1 (Identify the Problem or Issue): Through extensive literature reviews, our study aims to figure out the primary obstacles to NMAGs’ FP awareness in Bangladesh while highlighting specific definitions for focused policy responses.

Domain 2: Policy Analysis

*Step 2A (Identify and Describe Policy Options):* We reviewed the literature on adolescent SRH policies in Bangladesh and globally, examining existing programs, best practices, and environmental scans. This provided insights into effective strategies and highlighted gaps, ensuring a comprehensive view of potential improvements.

*Step 2B (Assess Policy Options*): A subgroup of the study team assessed each policy and program using a structured document review approach guided by four evaluation criteria drawn from the CDC framework, operationalized as follows: [1] *Effectiveness:* the extent to which the policy explicitly addresses the FP awareness needs of NMAGs and demonstrates evidence of reach or impact on this group; [2] *Efficiency:* the degree to which the policy achieves its stated objectives relative to resource inputs, including breadth of population coverage; [3] *Flexibility:* the degree to which the policy accommodates diverse and context-specific needs of NMAGs, including those in rural, low-income, or educationally disadvantaged settings; and [4] *Equity:* the extent to which the policy ensures equitable access to FP information for NMAGs as a distinct subgroup. Each criterion was assessed qualitatively based on a review of program documents, national strategy papers, donor evaluation reports, and peer-reviewed literature. Two authors (SN and MTA) conducted initial assessments independently; discrepancies were discussed and resolved by consensus with a third author (MMH). In the absence of a standardized scoring instrument validated for this context, assessments represent structured qualitative judgments rather than quantitative scores.

*Step 2C (Prioritize Policy Options):* We prioritized policy options for improving adolescent SRH and FP awareness in Bangladesh by assessing their effectiveness, feasibility, and potential impact, selecting the most promising for further development.

Domain 3: Strategy and Policy Development

*Step 3 (Develop a Strategy for Furthering Adoption of the Policy Solution):* After selecting the most effective policies, we developed a strategy to facilitate their enactment and implementation. This involved addressing operational issues, engaging and educating key stakeholders, and planning information dissemination to encourage adoption.

We developed a set of framing guided by the CDC framework (details in Supplemental Table 1) to systematically examine the extent to which each policy or program addresses FP awareness among NMAGs. The evaluative dimensions summarized in Supplemental Table 2 reflect these structured narrative judgments. Given that assessments were based exclusively on secondary document review and no primary data were collected for the policy analysis component, this section is best understood as structured narrative policy commentary informed by the CDC framework, rather than a formal quantitative policy evaluation.

### 2.6. Ethics approval and consent to participate

As this study used secondary data that are publicly available upon reasonable request, no Ethics Committee or Institutional Review Board approval was required. However, surveys involving human participants were reviewed and approved by the icddr,b ERC. Written informed consent to participate was provided by the participants’ legal guardians or next of kin.

## 3. Results

Among the surveyed NMAGs, roughly 25% were unaware of any modern FP methods. Specifically, 28% are unaware of oral contraceptive pills, 60% do not know about condoms, and 91% are not acquainted with emergency contraceptive pills.

### 3.1. Sample characteristics

The age distribution of the sample indicates that a significant proportion, approximately 75% of NMAGs, are aged between 15 and 17 years. Educational attainment among these individuals is relatively high, with about 92% having completed secondary or higher education (Table 2).

**Table 2:** Frequency and percentage of sociodemographic and information-access characteristics of never-married adolescent girls in Bangladesh, 2019–2020.

Covariate	Number of NMAGs	% of NMAGs
**Total:**	7742	
**Age**		
15-17	5,775	74.6
18-19	1,967	25.4
**Education**		
Class 0–5	614	7.9
Class 6–12	7,129	92.1
**Residence**		
Rural	5,264	68.0
Urban	2,478	32.0
**Division**		
Central	3,000	38.8
Western	2,313	29.9
Eastern	2,429	31.4
**Wealth index**		
Lowest	1,075	13.9
Second	1,490	19.2
Middle	1,696	21.9
Fourth	1,641	21.2
Highest	1,841	23.8
**Phone ownership/access**		
No ownership/access	481	6.2
Have ownership/access	7,261	93.8
**Internet**		
Do not use the internet weekly	6,037	78.0
Use the internet at least weekly	1,705	22.0
**Mass media access (TV, Radio, Newspaper)**		
No access to media	1,503	19.4
Access media at least once in week	6,239	80.6
**Exposure to program in last 3 months**		
No	6,453	83.4
Yes	1,289	16.7

Geographically, a majority (68%) of NMAGs reside in rural areas. Notably, a high percentage of the NMAGs (94%) had access to either a personal or household cell phone. However, internet accessibility remains limited, with only 22% of the NMAGs reporting access to the internet. Media exposure is high, with over 80% of NMAGs engaging with various media platforms at least once a week. On the other hand, only 17% of NMAGs had participated to any adolescent program in the last three months.

### 3.2. Bivariate analysis

Table 3 shows the bivariate distribution of awareness of modern FP methods across socio-demographic factors and exposure to adolescent program. Overall, every fourth adolescent is unaware of modern methods, while method-specific awareness is higher for condoms and ECPs. Significant differences are observed across most covariates.

**Table 3:** Percentages of never-married adolescent girls lacking awareness of modern family planning methods in Bangladesh, 2019–2020.

Covariates	Any modern method (%)	χ²	Pill (%)	χ²	Condom (%)	χ²	ECP (%)	χ²
Overall	24.93	–	28.08	–	60.15	–	90.68	–
Age		128.4**		138.7**		215.9**		86.2**
15–17	28.18		26.68		64.92		92.47	
18–19	15.38		16.14		46.13		85.42	
Education		59.5**		44.6**		119.2**		16.5**
Class 0–5	37.85		39.71		80.85		95.25	
Class 6–12	23.82		27.08		58.36		90.29	
Residence		2.1		0.32		48.4**		71.9**
Rural	25.41		28.28		62.80		92.60	
Urban	23.89		27.66		54.50		86.60	
Division		160.4**		120.0**		214.3**		11.0*
Central	25.28		28.89		58.39		90.29	
Western	16.56		20.29		50.75		89.55	
Eastern	32.46		34.50		71.27		92.24	
Wealth Index		17.0**		6.3		269.9**		196.9**
Poorest	25.51		27.87		71.24		94.26	
Poorer	25.84		27.75		67.87		95.73	
Middle	26.69		29.22		64.91		92.89	
Richer	25.89		29.52		56.84		89.95	
Richest	21.36		26.15		45.98		83.11	
Phone Ownership/Access		80.1**		—		95.3**		9.5*
No ownership/access	42.03		45.06		81.26		94.63	
Have ownership/access	23.79		45.06		58.75		90.42	
Internet		108.7**		77.8**		437.3**		167.5**
No access	27.65		30.48		66.33		92.95	
Access at least once/week	15.28		19.60		38.25		82.63	
Mass media		97.9**		79.5**		120.8**		27.4**
No access	34.84		37.36		72.61		94.20	
Access at least once/week	22.54		25.85		57.14		89.83	
Exposure to Program		63.8**		49.5**		74.6**		59.1**
No	26.68		29.69		62.29		91.81	
Yes	16.14		20.05		49.39		85.00	

**Note:** *Significant at p < 0.05, ** Significant at p < 0.001

Younger adolescents and those with lower educational attainment show comparatively higher levels of unawareness, indicating potential gaps in early and sustained information exposure. Among NMAGs aged 15–17 years, approximately 28% did not possess awareness of at least one modern FP method. Approximately 38% of NMAGs who had not completed secondary education were unaware of modern FP methods, compared to 24% among those who had completed secondary or higher education (Table 3). Access to communication resources appears important; adolescents without phone, internet, or media access tend to have higher levels of unawareness, suggesting the role of information access in shaping FP awareness. Almost one-third of NMAGs with access to a household phone were unaware of the modern FP method, compared to 42% among those without such access. Similarly, NMAGs with no internet and media access demonstrated lower FP awareness, at 42% and 28%, respectively. NMAGs who were not exposed to a program within the last three months reported lower awareness levels of FP (approximately 27%), compared to 16% among those who had been.

### 3.3 Regression output

***Awareness among NMAGs regarding pills, condoms, ECP, or any modern FP method (***Table 4***):*** NMAGs aged between 15 and 17 years had 52% lower odds of possessing awareness about FP compared to those aged 18–19 years. NMAGs who never attended secondary school had 35% lower odds of having FP awareness than those who completed secondary or higher education. The odds of having FP awareness was found to be 24% lower in the richest group compared to the poorest group. Additionally, being unable to access a phone (personal or household) was associated with lower odds of knowing at least one FP method. NMAGs who did not attend any adolescent program in the last three months demonstrated 38% lower odds of knowing at least one modern FP method compared to their counterparts.

**Table 4:** Adjusted odds ratio of knowing pills, condoms, ECP, or any modern FP among never-married adolescent girls in Bangladesh, 2019–2020.

	At least one modern method		Pill		Condom		ECP	
	aOR	[95% CI]	aOR	[95% CI]	aOR	[95% CI]	aOR	[95%CI]
**Age** (RC: 18–19)								
15-17	0.48^**^	[0.40,0.56]	0.48^**^	[0.41,0.56]	0.49^**^	[0.43,0.56]	0.54^**^	[0.45,0.65]
**Education** (RC: Class 6–12)								
Class 0–5	0.65^**^	[0.53,0.79]	0.68^**^	[0.56,0.83]	0.49^**^	[0.38,0.62]	0.67	[0.42,1.06]
**Residence** (RC: Urban)								
Rural	0.90	[0.74,1.08]	0.93	[0.78,1.11]	0.83^*^	[0.71,0.98]	0.72^**^	[0.59,0.88]
**Division** (RC: Western)								
Central	0.56^**^	[0.47,0.68]	0.62^**^	[0.52,0.73]	0.58^**^	[0.49,0.69]	0.70^**^	[0.57,0.86]
Eastern	0.39^**^	[0.32,0.48]	0.47^**^	[0.40,0.57]	0.30^**^	[0.25,0.36]	0.53^**^	[0.41,0.67]
**Wealth index** (RC: lowest)								
Second	0.85	[0.69,1.04]	0.88	[0.72,1.07]	1.04	[0.85,1.28]	0.67^*^	[0.46,0.97]
Middle	0.76^**^	[0.62,0.93]	0.76^**^	[0.63,0.93]	1.15	[0.95,1.38]	1.08	[0.76,1.54]
Fourth	0.70^**^	[0.57,0.88]	0.68^**^	[0.55,0.83]	1.45^**^	[1.18,1.78]	1.39	[0.95,2.04]
Highest	0.76^*^	[0.59,0.97]	0.69^**^	[0.55,0.86]	1.83^**^	[1.47,2.27]	2.06^**^	[1.40,3.03]
**Phone ownership/access** (RC: No ownership/access)								
No ownership/access	0.57^**^	[0.46,0.71]	0.57^**^	[0.45,0.71]	0.54^**^	[0.42,0.71]	0.94	[0.58,1.51]
**Internet** (RC: Access to internet)								
no access internet	0.54^**^	[0.44,0.65]	0.61^**^	[0.51,0.73]	0.41^**^	[0.35,0.48]	0.58^**^	[0.46,0.72]
**Media (TV, Radio, Newspaper)** (RC: access media at least once in week)								
No access to media	0.67^**^	[0.58,0.77]	0.67^**^	[0.59,0.77]	0.79^**^	[0.67,0.92]	0.95	[0.71,1.26]
**Exposure to program in last 3 months** (RC: Yes)								
No	0.62^**^	[0.51,0.75]	0.67^**^	[0.56,0.81]	0.73^**^	[0.63,0.85]	0.62^**^	[0.50,0.77]

**Note:** *Significant at p < 0.05, ** Significant at p < 0.001

Younger NMAGs had around 50% lower odds of knowing pills, condoms, and ECP compared to older NMAGs. Additionally, NMAGs who could not attend secondary school had lower odds of possessing awareness of pills and condoms compared to those who attended secondary school. NMAGs from rural areas exhibited 17–28% lower odds of knowing about condoms and ECP. Moreover, NMAGs residing in the eastern region had over 50% lower odds of knowing pills, condoms, and ECP compared to those in the western region. Awareness about pills significantly decreased with increasing wealth. However, awareness about condoms and ECP was higher among NMAGs from the upper wealth quintile.

### 3.4. Policy/Program Responses

Reviewing six national policies, programs, and strategies shows that existing FP initiatives largely overlook the specific FP-awareness needs of NMAGs—with the exception of the A2H program [36] —as summarized in Table 5.

**Table 5:** Analysis of family planning policies and programs for never-married adolescent girls in Bangladesh, 2012–2024.

Policy/ program name	Effectiveness	Efficiency	Flexibility	Equity
The Bangladesh National Adolescent Strategy (2020)	Rights-based, inclusive approach supporting overall adolescent development.	Limited; no targeted FP information for NMAGs risks uneven policy impact.	Flexible for adolescent needs; inadequate on NMAG-specific FP education.	Universal adolescent coverage in intent; FP knowledge gap for unmarried girls unaddressed.
National Adolescent Health Strategy (2017–2030)	Covers adolescent SRH broadly; limited on NMAG-specific FP.	Efficiency gaps likely from missing FP education for NMAGs.	Flexible management structures; no specific FP provisions.	NMAG-specific FP needs not addressed; unequal access to FP information.
The Advancing Adolescent Health (A2H) program (2016–2019)	Enhanced SRH and FP knowledge and service access for NMAGs and MAGs.	Constrained by short 2-year implementation period.	Highly flexible; strategies adapted to NMAG FP needs.	Equitable; NMAGs and MAGs received comparable FP access and knowledge.
Shukhi Jibon Project (2018–2023)	Improved FP and SRH services across platforms; no NMAG-specific interventions.	Reduced; NMAG-specific FP needs not directly targeted.	Limited; interventions not tailored to NMAGs.	Reduced; FP information provided but no services for NMAGs.
BALIKA program (2012–2016)	Reduced child marriage; improved education, health, and gender equity outcomes.	Limited on SRH outcomes due to absence of FP education.	Flexible across skill-building interventions; not for FP education.	Gains in education and livelihood equity; FP gap persists.
Engaging Volunteer Networks For Family Planning: Connecting Communities with Facilities (2015–2017)	High volunteer-led referral and FP method adoption rates.	Mixed; leveraged existing networks but constrained by non-remunerative model and uneven volunteer workloads.	Highly flexible; integrated with government services and adapted outreach to local needs.	Targeted hard-to-reach areas; access disparities persisted across implementation sites.

Note: Policy assessments reflect structured narrative judgments based on document review by the study team. Criteria definitions are provided in Methods Section 2.5.1. Assessments represent the consensus views of the authors and are not derived from a standardized scoring instrument.

The National Adolescent Health Strategy (NAS) 2017–2030 identifies key intervention areas including adolescent SRH, but it does not effectively cater to the unique FP needs of NMAGs [27].

The Bangladesh National Adolescent Strategy (2020) prioritizes equity, inclusion, and the developmental transitions of adolescents, implementing measures against gender discrimination and supporting adolescents with disabilities and those from minority groups [28]. However, it fails to provide specific guidelines for NMAGs, a crucial demographic requiring targeted policy attention.

The USAID-funded Shukhi Jibon project (2018–2024) worked to improve FP and SRH services broadly for those facing significant barriers, incorporating facility, community, school, and digital health-based activities for adolescents [37]. However, this initiative also did not specifically address the FP needs of NMAGs.

The only program that stands out is the A2H program, implemented by Plan International from January 2016 to January 2019 in Rangpur District. This program was specifically designed to enhance both the SRH and FP knowledge and service access for all adolescents, including NMAGs, assuming that improved access and knowledge within a supportive environment would yield significant benefits in the future [36].

The BALIKA program (2012–2016) aimed at delaying marriage for girls aged 12–18 through various empowering interventions, but did not incorporate FP education, further highlighting the gap in addressing the needs of NMAGs in national strategies [38].. Similarly, The Engaging Volunteer Networks for Family Planning program (2015–2017), aimed to enhance FP access in underserved areas of Bangladesh through local volunteer networks. While successful in broadening general access to FP methods like long-acting reversible contraceptives (LARCs) and permanent methods (PMs), the program did not offer specific interventions targeted at the unique needs of NMAGs [39].

## 4. Discussion

This study provides nationally representative evidence on FP awareness among NMAGs in Bangladesh, highlighting substantial socio-demographic disparities in awareness of modern contraceptive methods. Younger adolescents, particularly those aged 15–17 years, exhibited significantly lower awareness compared to older adolescents, suggesting an association between early adolescence and reduced exposure to FP information [40].

Educational attainment emerged as a strong correlate of FP awareness, with adolescents who had not completed secondary education demonstrating significantly lower awareness of contraceptive methods. This association underscores the importance of strengthening school-based SRH education, particularly at the secondary level, to support adolescents’ access to accurate FP information before transitioning into adulthood or marriage [41].

Access to mobile phones, the internet, and mass media was positively associated with FP awareness, suggesting a significant association between information channels and adolescents’ exposure to reproductive health information [42]. NMAGs without media access were significantly less likely to be aware of FP methods compared to those with access, underscoring the potential role of digital and mass media interventions in raising FP awareness among adolescents.

An interesting finding of this study is that adolescents from higher wealth quintiles showed lower overall awareness of at least one FP method, while demonstrating higher awareness of specific methods such as condoms and emergency contraceptive pills. We hypothesize that this contrasting pattern reflects differential exposure to method-specific information, for instance, adolescents from wealthier households may have greater access to media and digital platforms, which are often important sources of information on contraceptive methods [43]. However, our dataset does not include measures of media or information exposure, so this explanation remains untested. Further research is needed to confirm this mechanism.

NMAGs in the eastern region of Bangladesh (Chittagong, Sylhet) demonstrated lower awareness of FP compared with those in the western region (Rajshahi, Rangpur, Khulna). This variation maybe partially explained by regional differences in literacy and school dropout rates [44–46], which are known to shape access to health information at the population level. This pattern is also mirrored in the fertility rates, with the eastern region experiencing a higher fertility rate (2.78) compared to other regions in Bangladesh [47].

While overall FP awareness was similar for urban and rural areas, rural adolescents were less likely to know about condoms than their urban counterparts. FP has received substantial attention in various awareness programs from the government targating adolescents in rural areas [48], and NGOs also conduct various initiatives to raise FP awareness for rural residents [48,49]. Whereas in urban area, mobile apps and websites provide interactive learning environments for subjects including diverse contraceptive options, reproductive health, and safe practices [50,51]. The easy accessibility of information through mobile devices may be associated with greater awareness of FP practices among urban adolescents.

Exposure to adolescent-focused programs was significantly associated with higher FP awareness [52]. However, a large proportion of NMAGs reported no recent exposure to such programs, indicating substantial gaps in coverage. This suggests that existing initiatives may not be sufficiently reaching unmarried adolescents. The government and different NGOs are taking initiatives to spread FP awareness to adolescents through exposure programs, but most unmarried adolescents remain uncovered by those programs [53].

These findings are consistent with the policy analysis, which revealed that most national strategies and programs do not explicitly target NMAGs for FP-related interventions. The lack of tailored approaches may contribute to the observed disparities in awareness, particularly among younger, less educated, and socioeconomically disadvantaged adolescents.

### Policy suggestions

Our analysis of national policies and strategies in Bangladesh highlights significant gaps in addressing the FP and SRH needs of NMAGs. Existing national strategies like the National Adolescent Health Strategy (NAS) 2017–2030 and the Bangladesh National Adolescent Strategy (2020) rarely offer dedicated FP guidelines for NMAGs [53], despite emphasizing equity and inclusion.

The USAID-funded Shukhi Jibon project, while facilitating FP and SRH information, did not provide FP services specifically to NMAGs. Similarly, the BALIKA program, with its focus on delaying marriage, lacked dedicated FP education for this group. In contrast, the A2H program by Plan International has successfully integrated SRH and FP education for all adolescents, including NMAGs. Digital and community-based initiatives, such as Shukhi Jibon’s Kishore Batayan – Konnect platform and BRAC’s MAG clubs in urban slums show promising methods for spreading SRH information through digital outreach and community engagement [13,37], yet these need a greater focus on NMAGs.

To bridge these gaps, we recommend:

- Expanding the A2H model nationally to comprehensively meet NMAGs’ SRH and FP needs.
- Building on Shukhi Jibon’s experience in digital information delivery platforms such as *Kishore Batayan* and community-based initiatives like BRAC’s MAG clubs could incorporate targeted FP education for NMAGs.
- Developing and implementing dedicated policies that prioritize NMAGs’ unique needs, ensuring equitable access to accurate SRH and FP knowledge beyond basic awareness.

Overall, the findings highlight that while baseline awareness of FP methods exists among NMAGs, significant gaps remain, particularly in equitable access to information, underscoring the need for targeted interventions that move beyond awareness toward comprehensive knowledge.

### 4.1. Limitations and scope

This study has several limitations that should be considered when interpreting the findings. First, the measurement of FP awareness is based on whether respondents had “heard of” contraceptive methods. While this reflects recognition-based awareness and is widely used in large-scale surveys, it does not capture comprehensive knowledge, such as correct usage, effectiveness, or potential misconceptions regarding contraceptive methods. Therefore, the findings should be interpreted as representing baseline awareness rather than in-depth knowledge of FP methods.

Second, cultural and social norms in Bangladesh may influence responses to sensitive topics such as FP among NMAGs. This may lead to underreporting or socially desirable responses, potentially affecting data reliability.

Third, the survey lacks detailed information on contextual factors, such as the sources and quality of FP information accessed by adolescents, limiting the ability to assess how different information channels shape awareness.

Fourth, the policy analysis component of this study is based exclusively on secondary document review and should be interpreted as structured narrative commentary rather than a formal policy evaluation using a validated scoring instrument. The qualitative assessments of effectiveness, efficiency, flexibility, and equity presented in Table 5 represent the consensus judgments of the study team and may be subject to interpretation bias. Future analyses employing standardized assessment tools or independent multi-reviewer panels would strengthen the methodological rigor of policy appraisal.

Despite these limitations, the study provides nationally representative evidence on FP awareness among NMAGs in Bangladesh and highlights critical gaps that can inform future research and policy interventions.

## 5. Conclusion

FP awareness is essential for ensuring the well-being of SRH among NMAGs. The findings emphasize the need for targeted and inclusive interventions to address the socioeconomic disparities to improve FP awareness. The absence of tailored interventions and strategies for NMAGs in future programs further underscores the importance of developing focused initiatives. Combined efforts between the government and development partners are essential to ensure the SRHR of NMAGs in Bangladesh.

## Supporting information

S1 Table Framing questions for analysis of policies. (DOCX)

S2 Table CDC Policy Analytical Framework: Policy Analysis Table for Assessing Policy Options. (DOCX)
