# Supplementary material for: Family planning awareness among never-married adolescent girls in Bangladesh
Source: PLoS One. 2026 Oct 8;21(10):e0356778. doi: 10.1371/journal.pone.0356778 (PMC13648960; doi:10.1371/journal.pone.0356778)
Supplement: Supplementary file 1 [file pone.0356778.s005.pdf]

Family Planning Awareness among Never-Married Adolescent Girls in Bangladesh

Rebuttal letter

Academic editor:

1. Format the manuscript with headings and subheadings, apply Heading 1, Heading 2, Heading 3 styles appropriately.
2. Edit your manuscript carefully for spelling and grammar errors.
3. Format tables with no empty cells and include self-explanatory titles (answering *what*, *where*, and *when*).
4. Adjust spacing per journal guidelines.

Response: Thank you for these editorial suggestions. We have revised the manuscript accordingly by applying the appropriate heading styles, correcting spelling and grammatical errors, revising the tables, and adjusting the formatting and spacing to comply with the journal's guidelines.

Reviewers' comments:

Reviewer #2:

The manuscript addresses a genuinely important gap in adolescent reproductive health literature. The study makes three meaningful contributions: (1) establishing baseline national prevalence estimates for FP awareness among NMAGs; (2) quantifying sociodemographic disparities using rigorous multivariable analysis; and (3) systematically evaluating policy gaps through structured policy analysis. The use of BAHWS 2019-20 is appropriate for the research questions. The survey's national representativeness and adequate sample size (n=7,742 NMAGs) support generalizing inferences. However, the cross-sectional design fundamentally limits causal inference, a limitation the authors acknowledge but do not fully address in their interpretation

Comment 01. The quantitative results are generally well-interpreted, but several interpretive issues persist: The wealth paradox is the manuscript's most analytically challenging finding. The revised explanation (differential media access) is plausible but not directly tested. The authors should more explicitly acknowledge this as a hypothesis requiring further investigation rather than a settled interpretation.

Response 01. Thank you for this suggestion. We have revised the Discussion to explicitly state that the proposed explanation is hypothetical rather than a confirmed interpretation and clarified that the underlying mechanisms were not directly examined in this study (lines 330-335).

Comment 2. The Eastern region's lower awareness is attributed to literacy and dropout rate differences, with fertility rate data cited as corroborating evidence. However, this ecological reasoning (using population-level fertility rates to explain individual-level awareness) risks ecological fallacy. The authors should clarify that these are contextual, not causal, associations.

Response 2. We thank the reviewer for raising this important methodological point. We have revised the text to soften the conclusions (lines 336-339).

Comment 3. The abstract should specify the survey year (2019–20) to contextualize findings temporally.

Response 3. Thank you for your valuable comment. The survey year (2019–20) was already included in the abstract.

Comment 4. The manuscript has undergone language editing and is substantially improved. However, several issues remain:

Line 94: "Bangladesh is the 8th most largest populous lower-middle-income country" contains a grammatical error ("most largest" should be "most populous" or "largest").

Line 100: "Cass 0 to 5" appears in Table 2 (corrected to "Class 0 to 5" in the main text but persists in the table).
Line 272: "Exposer to program" should be "Exposure to program" (corrected in some places but not all).
Line 316: "loweress odds" should be "lower odds."

Response 4. Thank you for your careful reading of the manuscript and for identifying these errors. We have carefully reviewed the manuscript and corrected all of the issues you noted.
