# Supplementary material for: Family planning awareness among never-married adolescent girls in Bangladesh
Source: PLoS One. 2026 Oct 8;21(10):e0356778. doi: 10.1371/journal.pone.0356778 (PMC13648960; doi:10.1371/journal.pone.0356778)
Supplement: Supplementary file 2 [file pone.0356778.s001.docx]

**Supplementary Table 1:** Framing questions for analysis of family planning policies in Bangladesh

| **Framing questions** | |
| --- | --- |
| - What is the policy lever—is it legislative, administrative, regulatory, other? - What level of government or institution will implement this policy? - How does the policy work/operate? (e.g., is it mandatory? will enforcement be necessary?) - How is it funded? - Who is responsible for administering the policy? - What are the objectives of the policy? - Who are the target populations of the policy? - What is the legal landscape surrounding the policy (e.g., court rulings, constitutionality)? - What is the historical context (e.g., has the policy been developed based on an updated country context analysis and needs assessment)? - What is the value-added point of the policy? - What are the expected short, intermediate, and long-term outcomes? - What might be the unintended positive and negative consequences of the policy? | |
| **Criteria** | **Questions** |
| **Formulation stage** | 1. How does the policy address the problem? 2. What is the magnitude, reach, and distribution of benefit and burden? 3. What population will benefit? How much? When? 4. Will the policy impact health disparities/health equity? How? 5. Are the data updated based on which the policy is being formulated? 6. Are there gaps in the data/evidence-base? |
| **Followed up by a strategy** | 1. Has the policy been followed up by a strategy? 2. Is the strategy country-specific and culturally appropriate? |
| **Followed up by a plan of action** | 1. Has the strategy been followed up by an itemized plan of action? 2. Are the activities costed and budgeted for? 3. Have the responsible bodies been identified? 4. Has a definite timeline for each action item been identified? |
| **M&E** | 1. Are there indicators to assess the effectiveness of the policy points/activities 2. Are the policy/activity items flexible for revision? 3. Do the policy/activity items impact health disparities/health equity? 4. What are the institutional constraints? 5. Are each of the activities costed/budgeted? 6. Has the community accepted the activities? |
| **Policy revision** | 1. Is there any provision for policy revision? 2. Who will revise the policy? 3. Has there been any timeline fixed for assessment to determine if revision is required? |
