# Supplementary material for: Family planning awareness among never-married adolescent girls in Bangladesh
Source: PLoS One. 2026 Oct 8;21(10):e0356778. doi: 10.1371/journal.pone.0356778 (PMC13648960; doi:10.1371/journal.pone.0356778)
Supplement: Supplementary file 3 [file pone.0356778.s002.docx]

**Supplemental Table 2.** CDC Policy Analytical Framework for Assessing Family Planning Policy Options in Bangladesh

| **Criteria** | **Public Health Impact** | **Feasibility** | **Economic and Budgetary Impact** | |
| --- | --- | --- | --- | --- |
| **Scoring Definitions** | Low: small reach, effect size, and impact on disparate populations  Medium: small reach with large effect size or large reach with small effect size  High: large reach, effect size, and impact on disparate populations | Low: No/small likelihood of being enacted  Medium: Moderate likelihood of being enacted  High: High likelihood of being enacted | Budget  Less favorable: High costs to implement  Favorable: Moderate costs to implement  More favorable: Low costs to implement | Economic  Less favorable: costs are high relative to benefits  Favorable: costs are moderate relative to benefits (benefits justify costs)  More favorable: costs are low relative to benefits |
| **Policy 1** | ☐ Low  ☐ Medium  ☐ High  *Concerns about the amount or quality of data? (Yes / No)* | ☐ Low  ☐ Medium  ☐ High  *Concerns about the amount or quality of data? (Yes / No)* | ☐ Less favorable  ☐ Favorable  ☐ More favorable  *Concerns about the amount or quality of data? (Yes / No)* | ☐ Less favorable  ☐ Favorable  ☐ More favorable  *Concerns about the amount or quality of data? (Yes / No)* |
| **Policy 2** | ☐ Low  ☐ Medium  ☐ High  *Concerns about the amount or quality of data? (Yes / No)* | ☐ Low  ☐ Medium  ☐ High  *Concerns about the amount or quality of data? (Yes / No)* | ☐ Less favorable  ☐ Favorable  ☐ More favorable  *Concerns about the amount or quality of data? (Yes / No)* | ☐ Less favorable  ☐ Favorable  ☐ More favorable  *Concerns about the amount or quality of data? (Yes / No)* |
| **Policy 3** | ☐ Low  ☐ Medium  ☐ High  *Concerns about the amount or quality of data? (Yes / No)* | ☐ Low  ☐ Medium  ☐ High  *Concerns about the amount or quality of data? (Yes / No)* | ☐ Less favorable  ☐ Favorable  ☐ More favorable  *Concerns about the amount or quality of data? (Yes / No)* | ☐ Less favorable  ☐ Favorable  ☐ More favorable  *Concerns about the amount or quality of data? (Yes / No)* |

Note. Adapted from "CDC's Policy Analytical Framework," by the Centers for Disease Control and Prevention (CDC), 2024 (<https://www.cdc.gov/polaris/media/pdfs/2024/09/Table2.pdf>). T
