# Supplementary material for: Family planning awareness among never-married adolescent girls in Bangladesh
Source: PLoS One. 2026 Oct 8;21(10):e0356778. doi: 10.1371/journal.pone.0356778 (PMC13648960; doi:10.1371/journal.pone.0356778)
Supplement: Supplementary file 4 [file pone.0356778.s004.docx]

**ACADEMIC EDITOR:**

The manuscript contains editorial and formatting issues. Please address these concerns and format the manuscript according to the journal’s guidelines, including proper paragraphing, font size, and font style.

Tables should be placed within the manuscript rather than as annexes after the references.

Supplementary tables and other supporting materials should be listed at the end of the manuscript and cited appropriately within the text.

Additionally, please complete and upload the appropriate reporting checklist for your study design, available from the EQUATOR Network. Carefully edit the manuscript to resolve any language and editorial issues.

Reviewers' comments:

**Major Comments**

**Comment 1.** Conceptual Ambiguity: “Awareness” vs “Knowledge”

The manuscript uses the terms awareness, knowledge, and familiarity interchangeably. However, the outcome variable measures whether respondents have “heard of” contraceptive methods. This is recognition-based knowledge rather than comprehensive awareness. The authors should:

Clearly define the operational construct.
Justify why “heard of” is sufficient to represent awareness.
Discuss limitations of this proxy measure.
Without clarification, the conceptual framing is weak.

**Response 1:**

Thank you for this important observation. We agree that the terms “awareness,” “knowledge,” and “familiarity” were used interchangeably, which may lead to conceptual ambiguity.

In this study, the outcome variable is based on whether respondents have “heard of” specific contraceptive methods, which reflects recognition-based knowledge, a commonly used proxy for awareness in large-scale demographic surveys such as DHS and similar national datasets.

To address this concern, we have:

- Clearly defined the operational construct as recognition-based awareness of family planning methods in the Methods section.
- Standardized terminology throughout the manuscript, consistently using “awareness” to refer to “having heard of at least one modern FP method.”
- Justified the use of this measure by noting that in large-scale surveys, “heard of” is often used as an entry-level indicator of awareness, particularly in adolescent populations.
- Added a limitation acknowledging that this measure does not capture depth of knowledge, correct usage, or misconceptions.

**Comment 2.** Cross-sectional Design and Causal Language

Several sections imply causal relationships (e.g., program exposure improves knowledge; digital access promotes awareness). Given the cross-sectional design, such inferences are inappropriate.

The authors should:

Replace causal phrasing with associative language.

Explicitly acknowledge reverse causality as a limitation.

Clarify that program exposure and knowledge may be bidirectionally related.

**Response 2:** We thank the author for pointing this out. We have now revised all the interpretations more cautiously by replacing the causal tone.

**Comment 3.** Interpretation of Wealth Findings

The regression results indicate that higher wealth is associated with lower odds of knowing at least one method but higher odds of knowing condoms and ECPs (Table 4).

The discussion attempts to explain this using assumptions about premarital sexual activity among wealthier adolescents. These interpretations are speculative and unsupported by the dataset.

The authors should:

Avoid behavioral speculation not measured in the survey.

Provide alternative structural explanations (e.g., differential media consumption, method-specific messaging exposure).

**Response 3:** Thank you for this important comment. We agree that our earlier interpretations were assumption-based explanations that were not directly supported by the dataset. We have revised the discussion to remove these interpretations and instead focus on more plausible structural explanations, such as differential access to media and digital platforms. We have supported these explanations with relevant literature and acknowledged the limitations of the data in explaining these patterns.

**Comment 4.** Policy Analysis Lacks Methodological Transparency

The CDC policy analysis framework is described, but its application is insufficiently detailed.

Concerns:

No clear scoring rubric is presented.

It is unclear how “effectiveness,” “efficiency,” “flexibility,” and “equity” were operationalized.

No explanation of who conducted the assessment or whether multiple reviewers were involved.

Table 5 presents qualitative judgments without transparent evaluation criteria.

The policy component currently reads as narrative review rather than systematic policy analysis.

The authors must:

Provide explicit evaluation criteria.

Clarify analytic procedures.

Consider strengthening methodological rigor or reframing the section as narrative policy commentary.

**Response 4:** We agree with the reviewer that the methodological description of the policy analysis required greater clarity. We have revised the manuscript accordingly:

- Regarding the absence of a scoring rubric and unclear operationalization:

We have expanded Section 2.5.1 (Step 2B) to explicitly define the four evaluation domains: effectiveness (extent to which policies address FP awareness among NMAGs), efficiency (achievement of objectives relative to resource use and coverage), flexibility (ability to adapt to the specific needs of NMAGs), and equity (extent to which policies ensure inclusive access to FP information for NMAGs).

- Regarding the assessment process:

We have clarified that two authors (SN and MTA) independently conducted the initial assessments based on structured document review, with discrepancies resolved through discussion and consensus with a third author (MMH).

- Regarding transparency of Table 5:

We have added a methodological note in the table caption indicating that the findings represent structured qualitative judgments based on predefined criteria described in Section 2.5.1 and reflect consensus among the authors rather than outputs of a formal scoring system.

- Regarding the nature of the analysis:

We have revised Section 2.5.1 to clarify that the policy component represents a structured narrative policy analysis guided by the CDC framework, rather than a formal quantitative evaluation.

- Regarding limitations:

We have added a limitation in Section 4.1 acknowledging that the policy analysis relies on secondary document review, that qualitative assessments may be subject to interpretation bias, and that future studies using standardized tools or independent multi-reviewer panels could strengthen rigor.

We believe these revisions improve transparency and appropriately position the policy analysis within the scope of the study.

**Comment 5.** Statistical Reporting

While logistic regression is appropriate, additional clarification is needed:

Was complex survey design fully specified (cluster, strata, weights)?

Were multicollinearity diagnostics conducted?

Were model fit statistics assessed?

Why were certain reference categories selected?

Reporting should be strengthened to meet methodological standards.

**Response 5:** We thank the reviewer for this comment. Here is the response to the comments:

- **Complex survey design:** During the data analysis in STATA, we have used the **“svy”** command to adjust for cluster, strata, and weights, considering the complex nature of the survey.
- **Multicollinearity:** Multicollinearity was assessed using variance inflation factors (VIF) from an equivalent unweighted regression model; mean VIF values for Models 1–3 were 3.66, 2.57, and 2.57, indicating no problematic multicollinearity.
- **Model fit:** Model fit was evaluated using design-based F-statistics. All models were statistically significant (Model 1: F = 28.33; Model 2: F = 26.72; Model 3: F = 47.30; all p < 0.001), with improved fit observed in Model 3.
- Reference category: Reference groups were chosen based on the following principles:

1. **Policy relevance and conceptual baseline:** More advantaged or normative groups (e.g., older adolescents, higher education, urban residence, and access to media/internet) were used as reference categories to enable meaningful comparison with more vulnerable populations. This approach helps identify the extent to which disadvantaged groups lag, which is important for informing targeted policy and program interventions.
2. **Interpretability:** Using these groups as references facilitates clearer interpretation of odds ratios, particularly in identifying disparities in FP awareness across socio-demographic groups.

We have now added these to the method section **(lines 157-159).**

**Comment 6.** Literature Framing and Novelty Claim

The manuscript states that this is the “first study” assessing FP awareness among NMAGs nationally. This claim requires stronger justification. Existing literature on unmarried adolescent reproductive knowledge in Bangladesh should be more critically synthesized.

**Response 6:** Thank you for this valuable comment. We agree that the novelty claim required stronger justification and clearer positioning within the existing literature.

In response, we have revised the Introduction to include a more critical synthesis of prior studies on adolescent reproductive health knowledge in Bangladesh.

We have also moderated the novelty claim to: “to the best of our knowledge, no nationally representative study has examined FP method awareness and its sociodemographic correlates specifically among NMAGs in Bangladesh.” This wording is more accurate and cautious.

Moderate Comments
**Comment 7.** Language and Clarity

There are multiple grammatical and typographical errors (e.g., “Cass 0 to 5,” “Exposer to program”). Language requires professional editing.

Several sentences are repetitive or overly assertive.

**Response 7:** Thank you for this helpful comment. We have carefully reviewed the manuscript and corrected all grammatical and typographical errors, including those noted by the reviewer.

**Comment 8.** Ethical Statement

The manuscript states that no IRB approval was required due to secondary data use. However, the original survey’s ethical approval should be referenced explicitly.

**Response:** Thank you for this helpful comment. In the revised version, we explicitly mentioned the original survey’s ethical approval, along with the name of the approving organization and the consent-taking process.

**Comment 9. Tables**

Table formatting requires improvement.

Percentages in Table 3 need clearer presentation.

Table 5 is overly narrative and could be condensed.

**Response 9:** Thank you for this helpful comment.

**Table 3:** We have carefully revised Table 3 to enhance the clarity of the percentage presentation. Specifically, we have explicitly labeled all percentage columns (e.g., “(%)”), standardized formatting and alignment across rows, and improved visual consistency to facilitate easier interpretation. Importantly, while improving readability, we have retained the original structure of Table 3 to preserve the integrity and comparability of the descriptive results alongside the corresponding chi-square statistics.

**Table 5:** While the structure and content of Table 5 have not been modified, we have improved its clarity and interpretability through revisions described in Response 4. Specifically, we have clarified the evaluation criteria, assessment process, and the nature of the analysis in the Methods section and table caption. These additions provide the necessary context for understanding the qualitative judgments presented in Table 5.

We believe these revisions enhance the overall clarity of the tables while maintaining consistency with the study’s methodological approach.

**Reviewer #2:**

**Comment 1:** The policy analysis conclusions in table 5 rating programs on effectiveness, efficiency, flexibility, and equity are not traceable to any scoring data presented in the main manuscript, making those specific conclusions unverifiable from what has been submitted.

**Response 1:** Thank you for this important comment. We have clarified that the policy analysis is a structured qualitative assessment guided by the Centers for Disease Control and Prevention (CDC) framework and adapted for context, without a formal scoring system. The evaluation criteria (effectiveness, efficiency, flexibility, and equity) are now explicitly defined in Section 2.5.1, and the Table 5 caption has been revised to indicate that the ratings reflect qualitative judgments based on predefined criteria and document review, derived through author consensus.

These revisions improve the transparency and traceability of the policy analysis.

**Comment 2:** The Results section largely restates table values without adequate interpretation, and the integration between bivariate (Table 3) and multivariable (Table 4) findings is insufficiently developed.

**Response 2:** We thank the reviewer for the comment. In the result section of the revised version, we addressed the reviewers' comments by adding interpretations of the findings. We hope the revised version of the manuscript contains a better interpretation of the results.

**Comment 3:** The manuscript would benefit from thorough language editing, ideally by a fluent English speaker familiar with epidemiological writing.

**Response 3:** Thank you for your comment. We have gone through language editing and updated the writing accordingly.
