# Supplementary material for: Family planning awareness among never-married adolescent girls in Bangladesh
Source: PLoS One. 2026 Oct 8;21(10):e0356778. doi: 10.1371/journal.pone.0356778 (PMC13648960; doi:10.1371/journal.pone.0356778)
Supplement: Peer review history

# Peer review: Decision Letter 0

**DOI:** 10.1371/journal.pone.0356778.r001

**Authors:** Berhane Megerssa Ereso

24 Mar 2026

Dear Dr. Amin,

Thank you for submitting your manuscript to PLOS ONE. After careful consideration, we feel that it has merit but does not fully meet PLOS ONE’s publication criteria as it currently stands. Therefore, we invite you to submit a revised version of the manuscript that addresses the points raised during the review process.

**ACADEMIC EDITOR:**

Please submit your revised manuscript by %DATE-REVISION-DUE%. If you will need more time than this to complete your revisions, please reply to this message or contact the journal office at plosone@plos.org. When you're ready to submit your revision, log on to https://www.editorialmanager.com/pone/ and select the 'Submissions Needing Revision' folder to locate your manuscript file.

- A letter that responds to each point raised by the academic editor and reviewer(s). You should upload this letter as a separate file labeled 'Response to Reviewers'.
- A marked-up copy of your manuscript that highlights changes made to the original version. You should upload this as a separate file labeled 'Revised Manuscript with Track Changes'.
- An unmarked version of your revised paper without tracked changes. You should upload this as a separate file labeled 'Manuscript'.

If you would like to make changes to your financial disclosure, please include your updated statement in your cover letter. Guidelines for resubmitting your figure files are available below the reviewer comments at the end of this letter.

If applicable, we recommend that you deposit your laboratory protocols in protocols.io to enhance the reproducibility of your results. Protocols.io assigns your protocol its own identifier (DOI) so that it can be cited independently in the future. For instructions see: https://journals.plos.org/plosone/s/submission-guidelines#loc-laboratory-protocols. Additionally, PLOS ONE offers an option for publishing peer-reviewed Lab Protocol articles, which describe protocols hosted on protocols.io. Read more information on sharing protocols at https://plos.org/protocols?utm_medium=editorial-email&utm_source=authorletters&utm_campaign=protocols.

As the corresponding author, your ORCID iD is verified in the submission system and will appear in the published article. PLOS supports the use of ORCID, and we encourage all coauthors to register for an ORCID iD and use it as well. Please encourage your coauthors to verify their ORCID iD within the submission system before final acceptance, as unverified ORCID iDs will not appear in the published article. *Only*  the individual author can complete the verification step; PLOS staff *cannot*  verify ORCID iDs on behalf of authors.

We look forward to receiving your revised manuscript.

Kind regards,

Berhane Megerssa Ereso, PhD

Academic Editor

PLOS One

Journal Requirements:

When submitting your revision, we need you to address these additional requirements.

1. Please ensure that your manuscript meets PLOS ONE's style requirements, including those for file naming. The PLOS ONE style templates can be found at

https://journals.plos.org/plosone/s/file?id=wjVg/PLOSOne_formatting_sample_main_body.pdf and

https://journals.plos.org/plosone/s/file?id=ba62/PLOSOne_formatting_sample_title_authors_affiliations.pdf

2. Please note that your Data Availability Statement is currently missing the DOI/accession number of each dataset OR a direct link to access each database]. If your manuscript is accepted for publication, you will be asked to provide these details on a very short timeline. We therefore suggest that you provide this information now, though we will not hold up the peer review process if you are unable.

3. When completing the data availability statement of the submission form, you indicated that you will make your data available on acceptance. We strongly recommend all authors decide on a data sharing plan before acceptance, as the process can be lengthy and hold up publication timelines. Please note that, though access restrictions are acceptable now, your entire data will need to be made freely accessible if your manuscript is accepted for publication. This policy applies to all data except where public deposition would breach compliance with the protocol approved by your research ethics board. If you are unable to adhere to our open data policy, please kindly revise your statement to explain your reasoning and we will seek the editor's input on an exemption. Please be assured that, once you have provided your new statement, the assessment of your exemption will not hold up the peer review process.

4. PLOS requires an ORCID iD for the corresponding author in Editorial Manager on papers submitted after December 6th, 2016. Please ensure that you have an ORCID iD and that it is validated in Editorial Manager. To do this, go to ‘Update my Information’ (in the upper left-hand corner of the main menu), and click on the Fetch/Validate link next to the ORCID field. This will take you to the ORCID site and allow you to create a new iD or authenticate a pre-existing iD in Editorial Manager.

5. Please upload a copy of Supporting Information Figure/Table/etc. Supplemental Figure 1. which you refer to in your text on page 10.

6. Please include captions for your Supporting Information files at the end of your manuscript, and update any in-text citations to match accordingly. Please see our Supporting Information guidelines for more information: http://journals.plos.org/plosone/s/supporting-information.

7. If the reviewer comments include a recommendation to cite specific previously published works, please review and evaluate these publications to determine whether they are relevant and should be cited. There is no requirement to cite these works unless the editor has indicated otherwise.

Additional Editor Comments (if provided):

Dear Author,

Thank you for submitting you manuscript to the PLOS ONE

The manuscript contains editorial and formatting issues. Please address these concerns and format the manuscript according to the journal’s guidelines, including proper paragraphing, font size, and font style. Tables should be placed within the manuscript rather than as annexes after the references. Supplementary tables and other supporting materials should be listed at the end of the manuscript and cited appropriately within the text. Additionally, please complete and upload the appropriate reporting checklist for your study design, available from the EQUATOR Network. Carefully edit the manuscript to resolve any language and editorial issues.

[Note: HTML markup is below. Please do not edit.]

Reviewers' comments:

Reviewer's Responses to Questions

**Comments to the Author**

1. Is the manuscript technically sound, and do the data support the conclusions?

Reviewer #1: Yes

Reviewer #2: Partly

**********

2. Has the statistical analysis been performed appropriately and rigorously?

Reviewer #1: Yes

Reviewer #2: No

**********

3. Have the authors made all data underlying the findings in their manuscript fully available?

The PLOS Data policy

Reviewer #1: Yes

Reviewer #2: Yes

**********

4. Is the manuscript presented in an intelligible fashion and written in standard English?

Reviewer #1: Yes

Reviewer #2: No

**********

Reviewer #1: Summary

This manuscript examines family planning (FP) awareness among never-married adolescent girls (NMAGs) in Bangladesh using nationally representative BAHWS 2019–20 data and supplements the analysis with a CDC-based policy review. The topic is relevant and policy-significant. However, important conceptual, methodological, and analytical weaknesses limit the robustness of the conclusions.

Substantial revision is required before the manuscript can be considered for publication.

Major Comments

1. Conceptual Ambiguity: “Awareness” vs “Knowledge”

The manuscript uses the terms awareness, knowledge, and familiarity interchangeably. However, the outcome variable measures whether respondents have “heard of” contraceptive methods. This is recognition-based knowledge rather than comprehensive awareness.

The authors should:

Clearly define the operational construct.

Justify why “heard of” is sufficient to represent awareness.

Discuss limitations of this proxy measure.

Without clarification, the conceptual framing is weak.

2. Cross-sectional Design and Causal Language

Several sections imply causal relationships (e.g., program exposure improves knowledge; digital access promotes awareness). Given the cross-sectional design, such inferences are inappropriate.

The authors should:

Replace causal phrasing with associative language.

Explicitly acknowledge reverse causality as a limitation.

Clarify that program exposure and knowledge may be bidirectionally related.

3. Interpretation of Wealth Findings

The regression results indicate that higher wealth is associated with lower odds of knowing at least one method but higher odds of knowing condoms and ECPs (Table 4).

The discussion attempts to explain this using assumptions about premarital sexual activity among wealthier adolescents. These interpretations are speculative and unsupported by the dataset.

The authors should:

Avoid behavioral speculation not measured in the survey.

Provide alternative structural explanations (e.g., differential media consumption, method-specific messaging exposure).

4. Policy Analysis Lacks Methodological Transparency

The CDC policy analysis framework is described, but its application is insufficiently detailed.

Concerns:

No clear scoring rubric is presented.

It is unclear how “effectiveness,” “efficiency,” “flexibility,” and “equity” were operationalized.

No explanation of who conducted the assessment or whether multiple reviewers were involved.

Table 5 presents qualitative judgments without transparent evaluation criteria.

The policy component currently reads as narrative review rather than systematic policy analysis.

The authors must:

Provide explicit evaluation criteria.

Clarify analytic procedures.

Consider strengthening methodological rigor or reframing the section as narrative policy commentary.

5. Statistical Reporting

While logistic regression is appropriate, additional clarification is needed:

Was complex survey design fully specified (cluster, strata, weights)?

Were multicollinearity diagnostics conducted?

Were model fit statistics assessed?

Why were certain reference categories selected?

Reporting should be strengthened to meet methodological standards.

6. Literature Framing and Novelty Claim

The manuscript states that this is the “first study” assessing FP awareness among NMAGs nationally. This claim requires stronger justification. Existing literature on unmarried adolescent reproductive knowledge in Bangladesh should be more critically synthesized.

Moderate Comments

7. Language and Clarity

There are multiple grammatical and typographical errors (e.g., “Cass 0 to 5,” “Exposer to program”). Language requires professional editing.

Several sentences are repetitive or overly assertive.

8. Ethical Statement

The manuscript states that no IRB approval was required due to secondary data use. However, the original survey’s ethical approval should be referenced explicitly.

9. Tables

Table formatting requires improvement.

Percentages in Table 3 need clearer presentation.

Table 5 is overly narrative and could be condensed

Reviewer #2: The policy analysis conclusions in table 5 rating programs on effectiveness, efficiency, flexibility, and equity are not traceable to any scoring data presented in the main manuscript, making those specific conclusions unverifiable from what has been submitted. The Results section largely restates table values without adequate interpretation, and the integration between bivariate (Table 3) and multivariable (Table 4) findings is insufficiently developed. The manuscript would benefit from thorough language editing, ideally by a fluent English speaker familiar with epidemiological writing

**********

what does this mean?). If published, this will include your full peer review and any attached files.

If you choose “no”, your identity will remain anonymous but your review may still be made public.

**Do you want your identity to be public for this peer review?**  For information about this choice, including consent withdrawal, please see our Privacy Policy

Reviewer #1: No

Reviewer #2: **Yes:**  Ebenezer Antwi Amankwaah

**********

[NOTE: If reviewer comments were submitted as an attachment file, they will be attached to this email and accessible via the submission site. Please log into your account, locate the manuscript record, and check for the action link "View Attachments". If this link does not appear, there are no attachment files.]

To ensure your figures meet our technical requirements, please review our figure guidelines: https://journals.plos.org/plosone/s/figures

You may also use PLOS’s free figure tool, NAAS, to help you prepare publication quality figures: https://journals.plos.org/plosone/s/figures#loc-tools-for-figure-preparation.

NAAS will assess whether your figures meet our technical requirements by comparing each figure against our figure specifications.

# Author comment: Author response to Decision Letter 1

**DOI:** 10.1371/journal.pone.0356778.r002

5 May 2026

ACADEMIC EDITOR:

The manuscript contains editorial and formatting issues. Please address these concerns and format the manuscript according to the journal’s guidelines, including proper paragraphing, font size, and font style.

Tables should be placed within the manuscript rather than as annexes after the references.

Supplementary tables and other supporting materials should be listed at the end of the manuscript and cited appropriately within the text.

Additionally, please complete and upload the appropriate reporting checklist for your study design, available from the EQUATOR Network. Carefully edit the manuscript to resolve any language and editorial issues.

Reviewers' comments:

Major Comments

Comment 1. Conceptual Ambiguity: “Awareness” vs “Knowledge”

The manuscript uses the terms awareness, knowledge, and familiarity interchangeably. However, the outcome variable measures whether respondents have “heard of” contraceptive methods. This is recognition-based knowledge rather than comprehensive awareness. The authors should:

Clearly define the operational construct.

Justify why “heard of” is sufficient to represent awareness.

Discuss limitations of this proxy measure.

Without clarification, the conceptual framing is weak.

Response 1:

Thank you for this important observation. We agree that the terms “awareness,” “knowledge,” and “familiarity” were used interchangeably, which may lead to conceptual ambiguity.

In this study, the outcome variable is based on whether respondents have “heard of” specific contraceptive methods, which reflects recognition-based knowledge, a commonly used proxy for awareness in large-scale demographic surveys such as DHS and similar national datasets.

To address this concern, we have:

• Clearly defined the operational construct as recognition-based awareness of family planning methods in the Methods section.

• Standardized terminology throughout the manuscript, consistently using “awareness” to refer to “having heard of at least one modern FP method.”

• Justified the use of this measure by noting that in large-scale surveys, “heard of” is often used as an entry-level indicator of awareness, particularly in adolescent populations.

• Added a limitation acknowledging that this measure does not capture depth of knowledge, correct usage, or misconceptions.

Comment 2. Cross-sectional Design and Causal Language

Several sections imply causal relationships (e.g., program exposure improves knowledge; digital access promotes awareness). Given the cross-sectional design, such inferences are inappropriate.

The authors should:

Replace causal phrasing with associative language.

Explicitly acknowledge reverse causality as a limitation.

Clarify that program exposure and knowledge may be bidirectionally related.

Response 2: We thank the author for pointing this out. We have now revised all the interpretations more cautiously by replacing the causal tone.

Comment 3. Interpretation of Wealth Findings

The regression results indicate that higher wealth is associated with lower odds of knowing at least one method but higher odds of knowing condoms and ECPs (Table 4).

The discussion attempts to explain this using assumptions about premarital sexual activity among wealthier adolescents. These interpretations are speculative and unsupported by the dataset.

The authors should:

Avoid behavioral speculation not measured in the survey.

Provide alternative structural explanations (e.g., differential media consumption, method-specific messaging exposure).

Response 3: Thank you for this important comment. We agree that our earlier interpretations were assumption-based explanations that were not directly supported by the dataset. We have revised the discussion to remove these interpretations and instead focus on more plausible structural explanations, such as differential access to media and digital platforms. We have supported these explanations with relevant literature and acknowledged the limitations of the data in explaining these patterns.

Comment 4. Policy Analysis Lacks Methodological Transparency

The CDC policy analysis framework is described, but its application is insufficiently detailed.

Concerns:

No clear scoring rubric is presented.

It is unclear how “effectiveness,” “efficiency,” “flexibility,” and “equity” were operationalized.

No explanation of who conducted the assessment or whether multiple reviewers were involved.

Table 5 presents qualitative judgments without transparent evaluation criteria.

The policy component currently reads as narrative review rather than systematic policy analysis.

The authors must:

Provide explicit evaluation criteria.

Clarify analytic procedures.

Consider strengthening methodological rigor or reframing the section as narrative policy commentary.

Response 4: We agree with the reviewer that the methodological description of the policy analysis required greater clarity. We have revised the manuscript accordingly:

• Regarding the absence of a scoring rubric and unclear operationalization:

We have expanded Section 2.5.1 (Step 2B) to explicitly define the four evaluation domains: effectiveness (extent to which policies address FP awareness among NMAGs), efficiency (achievement of objectives relative to resource use and coverage), flexibility (ability to adapt to the specific needs of NMAGs), and equity (extent to which policies ensure inclusive access to FP information for NMAGs).

• Regarding the assessment process:

We have clarified that two authors (SN and MTA) independently conducted the initial assessments based on structured document review, with discrepancies resolved through discussion and consensus with a third author (MMH).

• Regarding transparency of Table 5:

We have added a methodological note in the table caption indicating that the findings represent structured qualitative judgments based on predefined criteria described in Section 2.5.1 and reflect consensus among the authors rather than outputs of a formal scoring system.

• Regarding the nature of the analysis:

We have revised Section 2.5.1 to clarify that the policy component represents a structured narrative policy analysis guided by the CDC framework, rather than a formal quantitative evaluation.

• Regarding limitations:

We have added a limitation in Section 4.1 acknowledging that the policy analysis relies on secondary document review, that qualitative assessments may be subject to interpretation bias, and that future studies using standardized tools or independent multi-reviewer panels could strengthen rigor.

We believe these revisions improve transparency and appropriately position the policy analysis within the scope of the study.

Comment 5. Statistical Reporting

While logistic regression is appropriate, additional clarification is needed:

Was complex survey design fully specified (cluster, strata, weights)?

Were multicollinearity diagnostics conducted?

Were model fit statistics assessed?

Why were certain reference categories selected?

Reporting should be strengthened to meet methodological standards.

Response 5: We thank the reviewer for this comment. Here is the response to the comments:

• Complex survey design: During the data analysis in STATA, we have used the “svy” command to adjust for cluster, strata, and weights, considering the complex nature of the survey.

• Multicollinearity: Multicollinearity was assessed using variance inflation factors (VIF) from an equivalent unweighted regression model; mean VIF values for Models 1–3 were 3.66, 2.57, and 2.57, indicating no problematic multicollinearity.

• Model fit: Model fit was evaluated using design-based F-statistics. All models were statistically significant (Model 1: F = 28.33; Model 2: F = 26.72; Model 3: F = 47.30; all p < 0.001), with improved fit observed in Model 3.

• Reference category: Reference groups were chosen based on the following principles:

1. Policy relevance and conceptual baseline: More advantaged or normative groups (e.g., older adolescents, higher education, urban residence, and access to media/internet) were used as reference categories to enable meaningful comparison with more vulnerable populations. This approach helps identify the extent to which disadvantaged groups lag, which is important for informing targeted policy and program interventions.

2. Interpretability: Using these groups as references facilitates clearer interpretation of odds ratios, particularly in identifying disparities in FP awareness across socio-demographic groups.

We have now added these to the method section (lines 157-159).

Comment 6. Literature Framing and Novelty Claim

The manuscript states that this is the “first study” assessing FP awareness among NMAGs nationally. This claim requires stronger justification. Existing literature on unmarried adolescent reproductive knowledge in Bangladesh should be more critically synthesized.

Response 6: Thank you for this valuable comment. We agree that the novelty claim required stronger justification and clearer positioning within the existing literature.

In response, we have revised the Introduction to include a more critical synthesis of prior studies on adolescent reproductive health knowledge in Bangladesh.

We have also moderated the novelty claim to: “to the best of our knowledge, no nationally representative study has examined FP method awareness and its sociodemographic correlates specifically among NMAGs in Bangladesh.” This wording is more accurate and cautious.

Moderate Comments

Comment 7. Language and Clarity

There are multiple grammatical and typographical errors (e.g., “Cass 0 to 5,” “Exposer to program”). Language requires professional editing.

Several sentences are repetitive or overly assertive.

Response 7: Thank you for this helpful comment. We have carefully reviewed the manuscript and corrected all grammatical and typographical errors, including those noted by the reviewer.

Comment 8. Ethical Statement

The manuscript states that no IRB approval was required due to secondary data use. However, the original survey’s ethical approval should be referenced explicitly.

Response: Thank you for this helpful comment. In the revised version, we explicitly mentioned the original survey’s ethical approval, along with the name of the approving organization and the consent-taking process.

Comment 9. Tables

Table formatting requires improvement.

Percentages in Table 3 need clearer presentation.

Table 5 is overly narrative and could be condensed.

Response 9: Thank you for this helpful comment.

Table 3: We have carefully revised Table 3 to enhance the clarity of the percentage presentation. Specifically, we have explicitly labeled all percentage columns (e.g., “(%)”), standardized formatting and alignment across rows, and improved visual consistency to facilitate easier interpretation. Importantly, while improving readability, we have retained the original structure of Table 3 to preserve the integrity and comparability of the descriptive results alongside the corresponding chi-square statistics.

Table 5: While the structure and content of Table 5 have not been modified, we have improved its clarity and interpretability through revisions described in Response 4. Specifically, we have clarified the evaluation criteria, assessment process, and the nature of the analysis in the Methods section and table caption. These additions provide the necessary context for understanding the qualitative judgments presented in Table 5.

We believe these revisions enhance the overall clarity of the tables while maintaining consistency with the study’s methodological approach.

Reviewer #2:

Comment 1: The policy analysis conclusions in table 5 rating programs on effectiveness, efficiency, flexibility, and equity are not traceable to any scoring data presented in the main manuscript, making those specific conclusions unverifiable from what has been submitted.

Response 1: Thank you for this important comment. We have clarified that the policy analysis is a structured qualitative assessment guided by the Centers for Disease Control and Prevention (CDC) framework and adapted for context, without a formal scoring system. The evaluation criteria (effectiveness, efficiency, flexibility, and equity) are now explicitly defined in Section 2.5.1, and the Table 5 caption has been revised to indicate that the ratings reflect qualitative judgments based on predefined criteria and document review, derived through author consensus.

These revisions improve the transparency and traceability of the policy analysis.

Comment 2: The Results section largely restates table values without adequate interpretation, and the integration between bivariate (Table 3) and multivariable (Table 4) findings is insufficiently developed.

Response 2: We thank the reviewer for the comment. In the result section of the revised version, we addressed the reviewers' comments by adding interpretations of the findings. We hope the revised version of the manuscript contains a better interpretation of the results.

Comment 3: The manuscript would benefit from thorough language editing, ideally by a fluent English speaker familiar with epidemiological writing.

Response 3: Thank you for your comment. We have gone through language editing and updated the writing accordingly.

Attachment Submitted filename: Rebuttal Letter_29 April2026.docx

# Peer review: Decision Letter 1

**DOI:** 10.1371/journal.pone.0356778.r003

**Authors:** Berhane Megerssa Ereso

8 Jul 2026

Dear Dr. Amin,

Thank you for submitting your manuscript to PLOS ONE. After careful consideration, we feel that it has merit but does not fully meet PLOS ONE’s publication criteria as it currently stands. Therefore, we invite you to submit a revised version of the manuscript that addresses the points raised during the review process.

**ACADEMIC EDITOR:**

1. Format the manuscript with headings and subheadings, apply Heading 1, Heading 2, Heading 3 styles appropriately.
2. Edit your manuscript carefully for spelling and grammar errors.
3. Format tables with no empty cells and include self-explanatory titles (answering *what* , *where* , and *when* ).
4. Adjust spacing per journal guidelines.

plosone@plos.org. When you're ready to submit your revision, log on to https://www.editorialmanager.com/pone/ and select the 'Submissions Needing Revision' folder to locate your manuscript file.

- A letter that responds to each point raised by the academic editor and reviewer(s). You should upload this letter as a separate file labeled 'Response to Reviewers'.
- A marked-up copy of your manuscript that highlights changes made to the original version. You should upload this as a separate file labeled 'Revised Manuscript with Track Changes'.
- An unmarked version of your revised paper without tracked changes. You should upload this as a separate file labeled 'Manuscript'.

If you would like to make changes to your financial disclosure, please include your updated statement in your cover letter. Guidelines for resubmitting your figure files are available below the reviewer comments at the end of this letter.

If applicable, we recommend that you deposit your laboratory protocols in protocols.io to enhance the reproducibility of your results. Protocols.io assigns your protocol its own identifier (DOI) so that it can be cited independently in the future. For instructions see: https://journals.plos.org/plosone/s/submission-guidelines#loc-laboratory-protocols. Additionally, PLOS ONE offers an option for publishing peer-reviewed Lab Protocol articles, which describe protocols hosted on protocols.io. Read more information on sharing protocols at https://plos.org/protocols?utm_medium=editorial-email&utm_source=authorletters&utm_campaign=protocols.

As the corresponding author, your ORCID iD is verified in the submission system and will appear in the published article. PLOS supports the use of ORCID, and we encourage all coauthors to register for an ORCID iD and use it as well. Please encourage your coauthors to verify their ORCID iD within the submission system before final acceptance, as unverified ORCID iDs will not appear in the published article. *Only*  the individual author can complete the verification step; PLOS staff *cannot*  verify ORCID iDs on behalf of authors.

We look forward to receiving your revised manuscript.

Kind regards,

Berhane Megerssa Ereso, PhD

Academic Editor

PLOS One

Journal Requirements:

If the reviewer comments include a recommendation to cite specific previously published works, please review and evaluate these publications to determine whether they are relevant and should be cited. There is no requirement to cite these works unless the editor has indicated otherwise.

Please review your reference list to ensure that it is complete and correct. If you have cited papers that have been retracted, please include the rationale for doing so in the manuscript text, or remove these references and replace them with relevant current references. Any changes to the reference list should be mentioned in the rebuttal letter that accompanies your revised manuscript. If you need to cite a retracted article, indicate the article’s retracted status in the References list and also include a citation and full reference for the retraction notice.

[Note: HTML markup is below. Please do not edit.]

Reviewers' comments:

Reviewer's Responses to Questions

**Comments to the Author**

Reviewer #1: All comments have been addressed

Reviewer #2: (No Response)

**********

2. Is the manuscript technically sound, and do the data support the conclusions?

Reviewer #1: Yes

Reviewer #2: Yes

**********

3. Has the statistical analysis been performed appropriately and rigorously?

Reviewer #1: Yes

Reviewer #2: Yes

**********

4. Have the authors made all data underlying the findings in their manuscript fully available?

The PLOS Data policy

Reviewer #1: Yes

Reviewer #2: Yes

**********

5. Is the manuscript presented in an intelligible fashion and written in standard English?

Reviewer #1: Yes

Reviewer #2: Yes

**********

Reviewer #1: everything was checked and its according to the standard. good luck in your futre paper. it should be good to be published/

Reviewer #2: The manuscript addresses a genuinely important gap in adolescent reproductive health literature. The study makes three meaningful contributions: (1) establishing baseline national prevalence estimates for FP awareness among NMAGs; (2) quantifying sociodemographic disparities using rigorous multivariable analysis; and (3) systematically evaluating policy gaps through structured policy analysis.

The use of BAHWS 2019-20 is appropriate for the research questions. The survey's national representativeness and adequate sample size (n=7,742 NMAGs) support generalizing inferences. However, the cross-sectional design fundamentally limits causal inference, a limitation the authors acknowledge but do not fully address in their interpretation

The quantitative results are generally well-interpreted, but several interpretive issues persist: The wealth paradox is the manuscript's most analytically challenging finding. The revised explanation (differential media access) is plausible but not directly tested. The authors should more explicitly acknowledge this as a hypothesis requiring further investigation rather than a settled interpretation. The Eastern region's lower awareness is attributed to literacy and dropout rate differences, with fertility rate data cited as corroborating evidence. However, this ecological reasoning (using population-level fertility rates to explain individual-level awareness) risks ecological fallacy. The authors should clarify that these are contextual, not causal, associations.

The abstract should specify the survey year (2019–20) to contextualize findings temporally.

The manuscript has undergone language editing and is substantially improved. However, several issues remain:

Line 94: "Bangladesh is the 8th most largest populous lower-middle-income country" contains a grammatical error ("most largest" should be "most populous" or "largest").

Line 100: "Cass 0 to 5" appears in Table 2 (corrected to "Class 0 to 5" in the main text but persists in the table).

Line 272: "Exposer to program" should be "Exposure to program" (corrected in some places but not all).

Line 316: "loweress odds" should be "lower odds."

**********

what does this mean?). If published, this will include your full peer review and any attached files.

If you choose “no”, your identity will remain anonymous but your review may still be made public.

**Do you want your identity to be public for this peer review?**  For information about this choice, including consent withdrawal, please see our Privacy Policy

Reviewer #1: No

Reviewer #2: No

**********

[NOTE: If reviewer comments were submitted as an attachment file, they will be attached to this email and accessible via the submission site. Please log into your account, locate the manuscript record, and check for the action link "View Attachments". If this link does not appear, there are no attachment files.]

To ensure your figures meet our technical requirements, please review our figure guidelines: https://journals.plos.org/plosone/s/figures

You may also use PLOS’s free figure tool, NAAS, to help you prepare publication quality figures: https://journals.plos.org/plosone/s/figures#loc-tools-for-figure-preparation.

NAAS will assess whether your figures meet our technical requirements by comparing each figure against our figure specifications.

# Author comment: Author response to Decision Letter 2

**DOI:** 10.1371/journal.pone.0356778.r004

19 Jul 2026

Family Planning Awareness among Never-Married Adolescent Girls in Bangladesh

Rebuttal letter

Academic editor:

1. Format the manuscript with headings and subheadings, apply Heading 1, Heading 2, Heading 3 styles appropriately.

2. Edit your manuscript carefully for spelling and grammar errors.

3. Format tables with no empty cells and include self-explanatory titles (answering what, where, and when).

4. Adjust spacing per journal guidelines.

Response: Thank you for these editorial suggestions. We have revised the manuscript accordingly by applying the appropriate heading styles, correcting spelling and grammatical errors, revising the tables, and adjusting the formatting and spacing to comply with the journal's guidelines.

Reviewers' comments:

Reviewer #2:

The manuscript addresses a genuinely important gap in adolescent reproductive health literature. The study makes three meaningful contributions: (1) establishing baseline national prevalence estimates for FP awareness among NMAGs; (2) quantifying sociodemographic disparities using rigorous multivariable analysis; and (3) systematically evaluating policy gaps through structured policy analysis.

The use of BAHWS 2019-20 is appropriate for the research questions. The survey's national representativeness and adequate sample size (n=7,742 NMAGs) support generalizing inferences. However, the cross-sectional design fundamentally limits causal inference, a limitation the authors acknowledge but do not fully address in their interpretation

Comment 01. The quantitative results are generally well-interpreted, but several interpretive issues persist: The wealth paradox is the manuscript's most analytically challenging finding. The revised explanation (differential media access) is plausible but not directly tested. The authors should more explicitly acknowledge this as a hypothesis requiring further investigation rather than a settled interpretation.

Response 01. Thank you for this suggestion. We have revised the Discussion to explicitly state that the proposed explanation is hypothetical rather than a confirmed interpretation and clarified that the underlying mechanisms were not directly examined in this study (lines 330-335).

Comment 2. The Eastern region's lower awareness is attributed to literacy and dropout rate differences, with fertility rate data cited as corroborating evidence. However, this ecological reasoning (using population-level fertility rates to explain individual-level awareness) risks ecological fallacy. The authors should clarify that these are contextual, not causal, associations.

Response 2. We thank the reviewer for raising this important methodological point. We have revised the text to soften the conclusions (lines 336-339).

Comment 3. The abstract should specify the survey year (2019–20) to contextualize findings temporally.

Response 3. Thank you for your valuable comment. The survey year (2019–20) was already included in the abstract.

Comment 4. The manuscript has undergone language editing and is substantially improved. However, several issues remain:

Line 94: "Bangladesh is the 8th most largest populous lower-middle-income country" contains a grammatical error ("most largest" should be "most populous" or "largest").

Line 100: "Cass 0 to 5" appears in Table 2 (corrected to "Class 0 to 5" in the main text but persists in the table).

Line 272: "Exposer to program" should be "Exposure to program" (corrected in some places but not all).

Line 316: "loweress odds" should be "lower odds."

Response 4. Thank you for your careful reading of the manuscript and for identifying these errors. We have carefully reviewed the manuscript and corrected all of the issues you noted.

Attachment Submitted filename: Rebuttal Letter_09July2026.pdf

# Editor report: Decision Letter 2

**DOI:** 10.1371/journal.pone.0356778.r005

**Authors:** Berhane Megerssa Ereso

10 Aug 2026

Family Planning Awareness among Never-Married Adolescent Girls in Bangladesh

PONE-D-25-56968R2

Dear Dr. Amin,

We’re pleased to inform you that your manuscript has been judged scientifically suitable for publication and will be formally accepted for publication once it meets all outstanding technical requirements.

Within one week, you’ll receive an e-mail detailing the required amendments. When these have been addressed, you’ll receive a formal acceptance letter and your manuscript will be scheduled for publication.

An invoice will be generated when your article is formally accepted. Please note, if your institution has a publishing partnership with PLOS and your article meets the relevant criteria, all or part of your publication costs will be covered. Please make sure your user information is up-to-date by logging into Editorial Manager at Editorial Manager® and clicking the ‘Update My Information' link at the top of the page. For questions related to billing, please contact billing support.

If your institution or institutions have a press office, please notify them about your upcoming paper to help maximize its impact. If they’ll be preparing press materials, please inform our press team as soon as possible -- no later than 48 hours after receiving the formal acceptance. Your manuscript will remain under strict press embargo until 2 pm Eastern Time on the date of publication. For more information, please contact onepress@plos.org.

Kind regards,

Berhane Megerssa Ereso, PhD

Academic Editor

PLOS One

Additional Editor Comments (optional):

Dear Author,

Please ensure the table titles include the place and year.

Best regards,

Reviewers' comments:

# Editor report: Acceptance letter

**DOI:** 10.1371/journal.pone.0356778.r006

**Authors:** Berhane Megerssa Ereso

PONE-D-25-56968R2

PLOS One

Dear Dr. Amin,

I'm pleased to inform you that your manuscript has been deemed suitable for publication in PLOS One. Congratulations! Your manuscript is now being handed over to our production team.

At this stage, our production department will prepare your paper for publication. This includes ensuring the following:

* All references, tables, and figures are properly cited

* All relevant supporting information is included in the manuscript submission,

* There are no issues that prevent the paper from being properly typeset

You will receive further instructions from the production team, including instructions on how to review your proof when it is ready. Please keep in mind that we are working through a large volume of accepted articles, so please give us a few days to review your paper and let you know the next and final steps.

Lastly, if your institution or institutions have a press office, please let them know about your upcoming paper now to help maximize its impact. If they'll be preparing press materials, please inform our press team within the next 48 hours. Your manuscript will remain under strict press embargo until 2 pm Eastern Time on the date of publication. For more information, please contact onepress@plos.org.

You will receive an invoice from PLOS for your publication fee after your manuscript has reached the completed accept phase. If you receive an email requesting payment before acceptance or for any other service, this may be a phishing scheme. Learn how to identify phishing emails and protect your accounts at https://explore.plos.org/phishing.

If we can help with anything else, please email us at customercare@plos.org.

Thank you for submitting your work to PLOS One and supporting open access.

Kind regards,

PLOS One Editorial Office Staff

on behalf of

Dr. Berhane Megerssa Ereso

Academic Editor

PLOS One
